# Scientists’ warning on affluence

**DOI:** 10.1038/s41467-020-16941-y

**Published:** 2020-06-19

**Authors:** Thomas Wiedmann, Manfred Lenzen, Lorenz T. Keyßer, Julia K. Steinberger

**Affiliations:** 1 Sustainability Assessment Program, School of Civil and Environmental Engineering, UNSW Sydney, Sydney, NSW Australia; 2 ISA, School of Physics, The University of Sydney, Sydney, NSW Australia; 3 Institute for Environmental Decisions, Department of Environmental Systems Science, ETH Zürich, Zürich, Switzerland; 4 Sustainability Research Institute (SRI), School of Earth and Environment, University of Leeds, Leeds, UK

**Keywords:** Environmental impact, Climate-change mitigation, Sustainability, Economics, Society

## Abstract

For over half a century, worldwide growth in affluence has continuously increased resource use and pollutant emissions far more rapidly than these have been reduced through better technology. The affluent citizens of the world are responsible for most environmental impacts and are central to any future prospect of retreating to safer environmental conditions. We summarise the evidence and present possible solution approaches. Any transition towards sustainability can only be effective if far-reaching lifestyle changes complement technological advancements. However, existing societies, economies and cultures incite consumption expansion and the structural imperative for growth in competitive market economies inhibits necessary societal change.

## Abstract (web summary)

Current environmental impact mitigation neglects over-consumption from affluent citizens as a primary driver. The authors highlight the role of bottom-up movements to overcome structural economic growth imperatives spurring consumption by changing structures and culture towards safe and just systems.

## Introduction

Recent scientists’ warnings confirm alarming trends of environmental degradation from human activity, leading to profound changes in essential life-sustaining functions of planet Earth^1–3^. The warnings surmise that humanity has failed to find lasting solutions to these changes that pose existential threats to natural systems, economies and societies and call for action by governments and individuals.

The warnings aptly describe the problems, identify population, economic growth and affluence as drivers of unsustainable trends and acknowledge that humanity needs to reassess the role of growth-oriented economies and the pursuit of affluence^1,2^. However, they fall short of clearly identifying the underlying forces of overconsumption and of spelling out the measures that are needed to tackle the overwhelming power of consumption and the economic growth paradigm^4^.

This perspective synthesises existing knowledge and recommendations from the scientific community. We provide evidence from the literature that consumption of affluent households worldwide is by far the strongest determinant and the strongest accelerator of increases of global environmental and social impacts. We describe the systemic drivers of affluent overconsumption and synthesise the literature that provides possible solutions by reforming or changing economic systems. These solution approaches range from reformist to radical ideas, including degrowth, eco-socialism and eco-anarchism. Based on these insights, we distil recommendations for further research in the final section.

## Affluence as a driver of environmental and social impacts

### The link between consumption and impacts

There exists a large body of literature in which the relationship between environmental, resource and social impacts on one hand, and possible explanatory variables on the other, is investigated. We review and summarise those studies that holistically assess the impact of human activities, in the sense that impacts are not restricted to the home, city, or territory of the individuals, but instead are counted irrespective of where they occur. Such an assessment perspective is usually referred to as consumption-based accounting, or footprinting^5^.

Allocating environmental impacts to consumers is consistent with the perspective that consumers are the ultimate drivers of production, with their purchasing decisions setting in motion a series of trade transactions and production activities, rippling along complex international supply-chain networks^5^. However, allocating impacts to consumers does not necessarily imply a systemic causal understanding of which actor should be held most responsible for these impacts. Responsibility may lie with the consumer or with an external actor, like the state, or in structural relations between actors. Scholars of sustainable consumption have shown that consumers often have little control over environmentally damaging decisions along supply chains^6^, however they often do have control over making a consumption decision in the first place. Whilst in Keynesian-type economics consumer demand drives production, Marxian political economics as well as environmental sociology views the economy as supply dominated^7^. In this paper, we highlight the measurement of environmental impacts of consumption, while noting that multiple actors bear responsibility.

Holistic studies of the environmental or social consequences of consumption usually involve the use of life-cycle assessment or input-output analysis that do not only account for direct (on-site, within-territory) but importantly also include indirect impacts occurring along global and complete supply chains^8,9^. The use of such methods is important, because failing to detect the outsourcing of indirect impacts (also called spill overs or leakage) has the potential to seriously undermine global environmental abatement efforts, e.g. on climate change^10^.

A significant proportionality between consumption and impact exists for a large range of environmental, resource and social indicators. The implications of consumption on scarce energy resources emerged already in the 1970s and was confirmed by many consumption-based analyses on indicators as varied as CO_2_ emissions, raw materials, air pollution, biodiversity, nitrogen emissions, scarce water use or energy^5,11^. Many of these studies employed multiple regression or similar techniques, yielding clear evidence for our first finding: that consumption is by far the strongest determinant of global impacts, dwarfing other socio-economic–demographic factors such as age, household size, qualification or dwelling structure^12–15^. Whilst the strength of the proportionality between consumption and impact decreases slightly towards higher incomes (measured by so-called elasticities), consumption was found to be a consistently positive driver. In other words, the impact intensity of consumption decreases, but absolute impacts increase towards higher consumption. Absolute decoupling, let alone an inverted-U-type Kuznets relationship, does not occur from a consumption-based accounting perspective^11,16,17^.

For some social indicators, causal associations between consumption and impact are weak or non-existent. For example, withdrawing consumption from countries with unequal wages, child labour, corruption or severe occupational hazards may not influence those conditions, and might even exacerbate social problems. Footprint studies on these indicators nevertheless characterise consumers of commodities from socially problematic origins as being implicated with detrimental impacts^9,18–20^.

### Trends

Many indicators of global environmental and social impacts have been monitored over time, and time series data exist^5^. Numerous global studies decomposing time series of footprints of consumption into drivers of trends have been carried out over the past decades, for example on greenhouse-gas emissions, energy use, water use, materials or mercury emissions. These studies routinely decompose global impact trends into effects due to changes in a number of factors, such as technology, the input structure of production, the product mix in consumer demand, the level of per-capita consumption or population^21^.

**Fig. 1:**
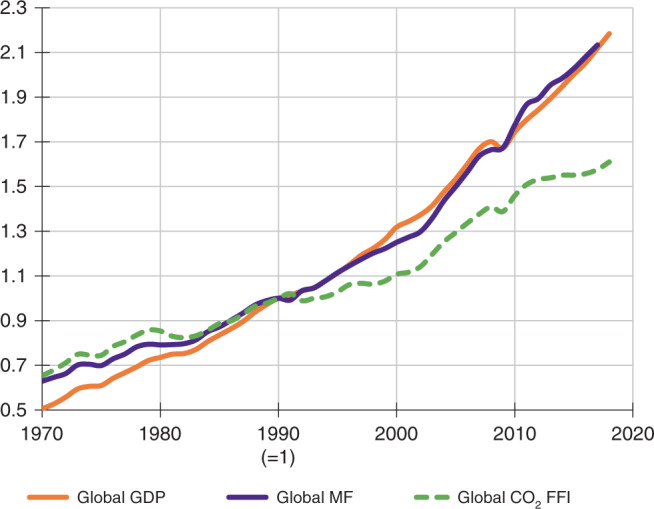
Relative change in main global economic and environmental indicators from 1970 to 2017. Shown is how the global material footprint (MF, equal to global raw material extraction) and global CO_2_ emissions from fossil-fuel combustion and industrial processes (CO_2_ FFI) changed compared with global GDP (constant 2010 USD). Indexed to 1 in 1990. Data sources: https://www.resourcepanel.org/global-material-flows-database, http://www.globalcarbonatlas.org and https://data.worldbank.org.

The majority of studies agree that by far the major drivers of global impacts are technological change and per-capita consumption^11^. Whilst the former acts as a more or less strong retardant, the latter is a strong accelerator of global environmental impact. Remarkably, consumption (and to a lesser extent population) growth have mostly outrun any beneficial effects of changes in technology over the past few decades. These results hold for the entire world^22,23^ as well as for numerous individual countries^11,24–26^. Figure 1 shows the example of changes in global-material footprint and greenhouse-gas emissions compared to GDP over time. The overwhelming evidence from decomposition studies is that globally, burgeoning consumption has diminished or cancelled out any gains brought about by technological change aimed at reducing environmental impact^11^.

Furthermore, low-income groups are rapidly occupying middle- and high-income brackets around the world. This can potentially further exacerbate the impacts of mobility-related consumption, which has been shown to disproportionately increase with income (i.e. the elasticity is larger than one^27^). This means that if consumption is not addressed in future efforts for mitigating environmental impact, technological solutions will face an uphill battle, in that they not only have to bring about reductions of impact but will also need to counteract the effects of growing consumption and affluence^28,29^.

To avoid further deterioration and irreversible damage to natural and societal systems, there will need to be a global and rapid decoupling of detrimental impacts from economic activity. Whilst a number of countries in the global North have recently managed to reduce greenhouse-gas emissions while still growing their economies^30^, it is highly unlikely that such decoupling will occur more widely in the near future, rapidly enough at global scale and for other environmental impacts^11,17^. This is because renewable energy, electrification, carbon-capturing technologies and even services all have resource requirements, mostly in the form of metals, concrete and land^31^. Rising energy demand and costs of resource extraction, technical limitations and rebound effects aggravate the problem^28,32,33^. It has therefore been argued that “policy makers have to acknowledge the fact that addressing environmental breakdown may require a direct downscaling of economic production and consumption in the wealthiest countries”^17,p.5^. We will address this argument in the section on systemic drivers and possible solutions.

### International disparities

In what follows, we will explain why we characterise consumption as affluence. Inequality is commonly described by the Gini index, with 0 characterising total equality (all individuals equal) and 100 representing total inequality (one individual owning everything). World countries’ Gini indices of income inequality range between 25 (Scandinavia) and 63 (Southern Africa)^34^. The world’s Gini index of income inequality is around 75, higher than the corresponding index of any national population. Simply put, the world as a whole is more unequal than any individual country.

Since income is strongly linked with consumption, and consumption is in turn linked with impact (see previous section), we can expect existing income inequalities to translate into equally significant impact inequalities. Indeed, environmental, resource and social impacts are exerted unequally across the world population. Teixido-Figueras et al.^35^ report that international Gini coefficients for CO_2_ emissions, material consumption and net primary productivity (both measured from a production and consumption perspective) range between 35 and 60. These values mean that the world’s top 10% of income earners are responsible for between 25 and 43% of environmental impact. In contrast, the world’s bottom 10% income earners exert only around 3–5% of environmental impact^35^. These findings mean that environmental impact is to a large extent caused and driven by the world’s rich citizens^36^. Considering that the lifestyles of wealthy citizens are characterised by an abundance of choice, convenience and comfort, we argue that the determinant and driver we have referred to in previous sections as consumption, is more aptly labelled as affluence.

Teixido-Figueras et al.^35^ also find that carbon emissions and material use are globally more unequally distributed when accounted for as footprints. In contrast to territorial allocations, footprints attribute environmental burdens to the final consumer, no matter where the initial environmental pressure has occurred. Here, international trade is responsible for shifting burdens from mostly low-income developing-world producers to high-income developed-world consumers^37^. This phenomenon of outsourcing appears to exacerbate global disparities, at least in carbon emissions and material use contexts.

## Systemic drivers and possible solutions

As the previous section shows, there is a positive relationship between biophysical resource use and affluence, as defined by income. Adding to this, the most affluent groups have higher incomes than expenditure, and their saving and investing leads to substantial additional environmental impact^38^. Therefore, and due to significant inter- and intra-national wealth and income inequality^36,39^, we differentiate between globally affluent groups, such as the European Union, and the most wealthy and affluent groups within countries, e.g. the <1–10% richest income segments^36^. As quantitative research^36,40,41^ shows, highly affluent consumers drive biophysical resource use (a) directly through high consumption, (b) as members of powerful factions of the capitalist class and (c) through driving consumption norms across the population. The next sections focus on affluent groups globally and on the intra-nationally most wealthy and affluent segments (hereafter called super-affluent).

### Reducing overconsumption

Since the level of consumption determines total impacts, affluence needs to be addressed by *reducing* consumption, not just greening it^17,28,29^. It is clear that prevailing capitalist, growth-driven economic systems have not only increased affluence since World War II, but have led to enormous increases in inequality, financial instability, resource consumption and environmental pressures on vital earth support systems^42^. A suitable concept to address the ecological dimension is the widely established avoid-shift-improve framework outlined by Creutzig et al.^43^. Its focus on the end-use service, such as mobility, nutrition or shelter, allows for a multi-dimensional analysis of potential impact reductions beyond sole technological change. This analysis can be directed at human need satisfaction or decent living standards—an alternative perspective put forward for curbing environmental crises^44,45^. Crucially, this perspective allows us to consider different provisioning systems (e.g. states, markets, communities and households) and to differentiate between superfluous consumption, which is consumption that does not contribute to needs satisfaction, and necessary consumption which can be related to satisfying human needs. It remains important to acknowledge the complexities surrounding this distinction, as touched upon in the sections on growth imperatives below. Still, empirically, human needs satisfaction shows rapidly diminishing returns with overall consumption^45,46^.

**Fig. 2:**
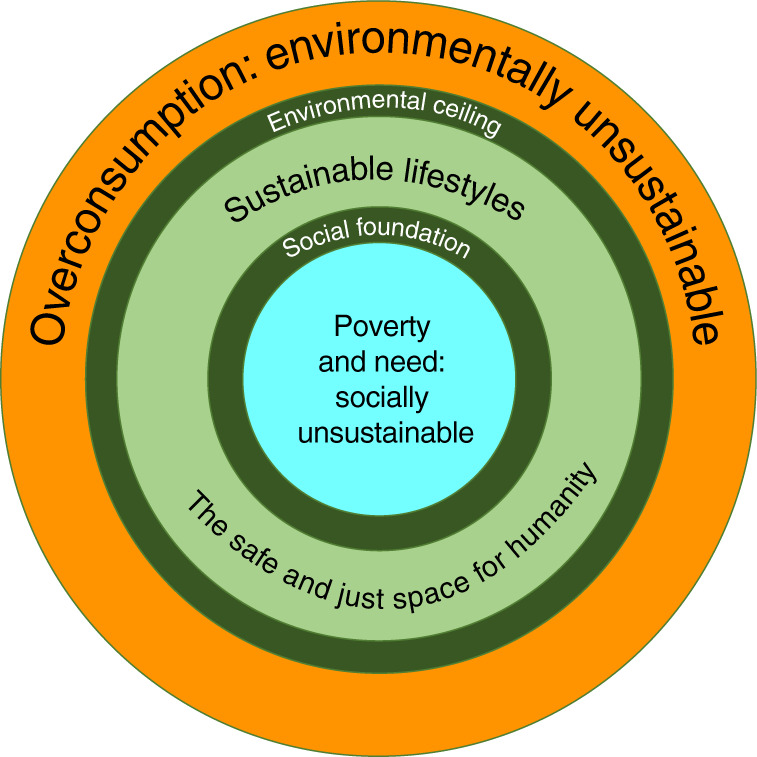
The safe and just space for humanity. Sustainable lifestyles are situated between an upper limit of permissible use (“Environmental ceiling”) and a lower limit of necessary use of environmental resources (“Social foundation”) (figures from ref. ^49^ and ref. ^84^ combined and adapted).

As implied by the previous section on affluence as a driver, the strongest pillar of the necessary transformation is to avoid or to reduce consumption until the remaining consumption level falls within planetary boundaries, while fulfilling human needs^17,28,46^. Avoiding consumption means not consuming certain goods and services, from living space (overly large homes, secondary residences of the wealthy) to oversized vehicles, environmentally damaging and wasteful food, leisure patterns and work patterns involving driving and flying^47^. This implies reducing expenditure and wealth along ‘sustainable consumption corridors’, i.e. minimum and maximum consumption standards^48,49^ (Fig. 2). On the technological side, reducing the need for consumption can be facilitated by changes such as increasing lifespans of goods, telecommunication instead of physical travel, sharing and repairing instead of buying new, and house retrofitting^43^.

However, the other two pillars of shift and improve are still vital to achieve the socio-ecological transformation^46^. Consumption patterns still need to be shifted away from resource and carbon-intensive goods and services, e.g. mobility from cars and airplanes to public buses and trains, biking or walking, heating from oil heating to heat pumps, nutrition—where possible—from animal to seasonal plant-based products^43,46^. In some cases this includes a shift from high- to low-tech (with many low-tech alternatives being less energy intense than high-tech equivalents, e.g. clothes line vs. dryer) and from global to local^47^. In parallel, also the resource and carbon intensity of consumption needs to be decreased, e.g. by expanding renewable energy, electrifying cars and public transport and increasing energy and material efficiency^43,46^.

The avoid-shift-improve framework, coherently applied with a dominant avoid and strong shift, implies the adoption of less affluent, simpler and sufficiency-oriented lifestyles to address overconsumption—consuming better but less^46,47,49,50^. This also includes addressing socially unsustainable underconsumption in impoverished communities in both less affluent and affluent countries, where enough and better is needed to achieve a more equal distribution of wealth and guarantee a minimum level of prosperity to overcome poverty^48,49^. Thus, establishing a floor-and-ceiling strategy of sustainable consumption corridors is necessary^48,49^ (Fig. 2).

It is well established that at least in the affluent countries a persistent, deep and widespread reduction of consumption and production would reduce economic growth as measured by gross domestic product (GDP)^51,52^. Estimates of the needed reduction of resource and energy use in affluent countries, resulting in a concomitant decrease in GDP of similar magnitude, range from 40 to 90%^53,54^. Bottom-up studies, such as from Rao et al.^55^ show that decent living standards could be maintained in India, Brazil and South Africa with around 90% less per-capita energy use than currently consumed in affluent countries. Trainer^56^, for Australia, and Lockyer^57^, for the USA, find similar possible reductions. In current capitalist economies such reduction pathways would imply widespread economic recession with a cascade of currently socially detrimental effects, such as a collapse of the stock market, unemployment, firm bankruptcies and lack of credit^50,58^. The question then becomes how such a reduction in consumption and production can be made socially sustainable, safeguarding human needs and social function^50,59^ However, to address this question, we first need to understand the various growth imperatives of capitalist social and economic systems and the role of the super-affluent segments of society^60^.

### Super-affluent consumers and growth imperatives

Growth imperatives are active at multiple levels, making the pursuit of economic growth (net investment, i.e. investment above depreciation) a necessity for different actors and leading to social and economic instability in the absence of it^7,52,60^. Following a Marxian perspective as put forward by Pirgmaier and Steinberger^61^, growth imperatives can be attributed to capitalism as the currently dominant socio-economic system in affluent countries^7,51,62^, although this is debated by other scholars^52^. To structure this topic, we will discuss different affected actors separately, namely corporations, states and individuals, following Richters and Siemoneit^60^. Most importantly, we address the role of the super-affluent consumers within a society, which overlap with powerful fractions of the capitalist class. From a Marxian perspective, this social class is structurally defined by its position in the capitalist production process, as financially tied with the function of capital^63^. In capitalism, workers are separated from the means of production, implying that they must compete in labour markets to sell their labour power to capitalists in order to earn a living.

Even though some small- and medium-sized businesses manage to refrain from pursuing growth, e.g. due to a low competition intensity in niche markets, or lack of financial debt imperatives, this cannot be said for most firms^64^. In capitalism, firms need to compete in the market, leading to a necessity to reinvest profits into more efficient production processes to minimise costs (e.g. through replacing human labour power with machines and positive returns to scale), innovation of new products and/or advertising to convince consumers to buy more^7,61,62^. As a result, the average energy intensity of labour is now twice as high as in 1950^60^. As long as a firm has a competitive advantage, there is a strong incentive to sell as much as possible. Financial markets are crucial to enable this constant expansion by providing (interest-bearing) capital and channelling it where it is most profitable^58,61,63^. If a firm fails to stay competitive, it either goes bankrupt or is taken over by a more successful business. Under normal economic conditions, this capitalist competition is expected to lead to aggregate growth dynamics^7,62,63,65^.

However, two factors exist that further strengthen this growth dynamic^60^. Firstly, if labour productivity continuously rises, then aggregate economic growth becomes necessary to keep employment constant, otherwise technological unemployment results. This creates one of the imperatives for capitalist states to foster aggregate growth, since with worsening economic conditions and high unemployment, tax revenues shrink, e.g. from labour and value-added taxes, while social security expenditures rise^60,62^. Adding to this, states compete with other states geopolitically and in providing favourable conditions for capital, while capitalists have the resources to influence political decisions in their favour. If economic conditions are expected to deteriorate, e.g. due to unplanned recession or progressive political change, firms can threaten capital flight, financial markets react and investor as well as consumer confidence shrink^51,58,60^. Secondly, consumers usually increase their consumption in tune with increasing production^60^. This process can be at least in part explained by substantial advertising efforts by firms^47,52,66^. However, further mechanisms are at play as explained further below.

Following this analysis, it is not surprising that the growth paradigm is hegemonic, i.e. the perception that economic growth solves all kinds of societal problems, that it equals progress, power and welfare and that it can be made practically endless through some form of supposedly green or sustainable growth^59^. Taken together, the described dynamics create multiple dependencies of workers, firms and states on a well-functioning capital accumulation and thus wield more material, institutional and discursive power (e.g. for political lobbying) to capitalists who are usually the most affluent consumers^61,67^. Even if different fractions of the capitalist class have manifold and competing interests which need to be constantly renegotiated, there is a common interest in maintaining the capitalist system and favourable conditions for capital accumulation, e.g. through aggregate growth and high consumption^51,62^. How this political corruption by the super-affluent plays out in practice is well documented, e.g. for the meat industry in Denmark^6^.

### Super-affluent consumers drive consumption norms

Growth imperatives and drivers (with the latter describing less coercive mechanisms to increase consumption) can also be active at the individual level. In this case, the level of consumption can serve as a proxy^47,60,68^. To start with, individual consumption decisions are not made in a vacuum, but are shaped by surrounding (physical and social) structures and provisioning systems^47,61,69^. Sanne^66^ and Alexander^47^ discuss several structural barriers to sufficiency-oriented lifestyles, locking in high consumption. These include lack of suitable housing, insufficient options for socialising, employment, transport and information, as well as high exposure to consumer temptations. Often, these conditions are deliberately fostered by states and also capitalists (the latter overlapping with super-affluent consumers and having disproportionate influence on states) to increase consumption^61,66^.

Further active mechanisms to spur growth include positional and efficiency consumption, which contribute to an increase in consumption overall^52,60,68,70^. After basic material needs are satisfied, an increasing proportion of consumption is directed at positional goods^52,70^. The defining feature of these goods is that they are expensive and signify social status. Access to them depends on the income relative to others. Status matters, since empirical studies show that currently relative income is one of the strongest determinants of individual happiness^52^. In the aggregate however, the pursuit of positional consumption, driven by super-affluent consumers and high inequalities, likely resembles a zero-sum game with respect to societal wellbeing^70,71^. With every actor striving to increase their position relative to their peers, the average consumption level rises and thus even more expensive positional goods become necessary, while the societal wellbeing level stagnates^42,71^. This is supported by a large body of empirical research, showing that an individual’s happiness correlates positively with their own income but negatively with the peer group’s income^71^ and that unequal access to positional goods fosters rising consumption^52^. This endless process is a core part of capitalism as it keeps social momentum and consumption high with affluent consumers driving aspirations and hopes of social ascent in low-affluence segments^70,72^. The positional consumption behaviour of the super-affluent thus drives consumption norms across the population, for instance through their excessive air travel, as documented by Gössling^73^.

Lastly, in capitalism, workers must compete against each other in the labour market in order to earn a living from capitalists^7,63^. Following Siemoneit^68^, this can lead to a similar imperative to net invest (increase the level of consumption/investment) as is observed with capitalists. In order to stay competitive, individuals are pushed to increase time and cost efficiency by investing in cars, kitchen appliances, computers and smartphones, by using social media and online trade etc. This efficiency consumption—effectively another facet of the rebound effect^38,47,68^—helps to manage high workloads, thus securing an income, while maintaining private life. This is often accompanied by trends of commodification^61^, understood as the marketisation of products and services which used to be provisioned through more time-intensive commons or reciprocal social arrangements, e.g. convenience food vs. cooking together. As in the food example^74^, this replacement of human labour with energy- and material-intensive industrial production typically increases environmental pressures^47,75^. Through these economic pressures, positive feedback loops and lock-ins are expected to emerge, since other consumers need to keep up with these investments or face disadvantages, e.g. when car or smartphone ownership become presupposed. Taken together with positional consumption, structural barriers to sufficiency and the substantial advertising efforts by capitalists, these mechanisms explain to a large extent why consumers seem so willing to increase their consumption in accordance with increasing production^60^.

### Solution approaches

In response to the aforementioned drivers of affluence, diverse solution approaches and strategies are being discussed^47,52,76^. We differentiate these as belonging to a more reformist and a more radical group (Table 1). This is based on the categorisation by Alexander and Rutherford^77^. All these approaches differ from the established green growth (ecomodernism) approach^28,78,79^, in that they at least adopt an agnostic, if not negative, position on the question whether or not GDP can be sufficiently decoupled from environmental impacts^28,52,78,80^. Hence, these approaches also differ from the Sustainable Development Goals (SDGs), since SDG 8 aims for continued global GDP growth of ~3% p.a., likely contradicting several other SDGs, e.g. SDG 12 and 13^81–83^. Further, the SDGs are not representing a theoretically coherent framework, since they are part of a deliberative process^45^, and sideline underlying power dynamics as well as interactions between injustices^83^. Nevertheless, approaches underpinned by multi-dimensional social wellbeing and environmental goals, such as Kate Raworth’s Doughnut Economics^84^, are strong alternatives to GDP-focused ones and may inspire transformative change in the context of the more reformist solution approaches outlined below. Importantly, the following discussion can only provide a rough overview of the respective approaches.

**Table 1:** Meta approaches for sustainable prosperity.

	Radical approaches		Reformist approaches	Green growth approach
Sub-group	Eco-socialism (incl. degrowth)	Eco-anarchism	A-growth, precautionary/pragmatic post-growth, steady-state economy, prosperity and managing without growth	Sustainable growth, ecological modernisation, decoupling
Key references	^47,50,51,59,65^	^54,87^	^42,52,80,85,86^	^28,78,79^
Key premise/principle/hypothesis/assumptions	• Decoupling is most likely not possible • Necessary changes are most likely not compatible with capitalism • The democratic state is expected to play a significant role in the transition and beyond, although grassroots movements are still important	• Decoupling is most likely not possible • Necessary changes are most likely not compatible with capitalism • The state is not expected to play a significant role in the transition. Instead, grassroots participatory-democratic movements are central in the transition and beyond	• Group 1: infinite growth on a finite planet (decoupling) is most likely not possible (Daly, Victor or Jackson) • Group 2: agnostic to growth; decoupling could still be possible; uncertainty (van den Bergh, Petschow et al.) • Necessary changes are compatible with centralised states and capitalism	• Economic growth can be decoupled from environmental impacts and is necessary to provide sustainable technical solutions. • Necessary changes are compatible with centralised states and capitalism
Goals/aspirations	Decouple wellbeing from GDP growth, shrink impacts and expect GDP shrinkage, increase social control over economy using the state	Decouple wellbeing from GDP growth, shrink impacts and expect GDP shrinkage, increase social control over economy without using the state	Decouple wellbeing from GDP growth, shrink impacts despite possible/likely GDP decrease	Maintain high economic growth and decrease impacts (decoupling)
Mechanisms	Focus on resource limits, system change and wellbeing	Focus on resource limits, system change and wellbeing	Focus on resource limits, reforms and wellbeing	Focus on resource efficiency, renewable energy and decoupling
Institutions/actors	Governments, civil society and grassroots initiatives, voters, scientists	Civil society and grassroots initiatives, scientists	Governments, civil society and grassroots initiatives, voters, scientists	Governments, financial institutions, voters, scientists,
Actions	Include strong limits and social justice in policies; Change economic structures, reform institutions and increase social control over economic actions; change lifestyles, consciousness and cultures through grassroots action	Change lifestyles, cultures and consciousness through grassroots action; Build alternative localised participatory-democratic economic system besides old one and remove barriers through cooperating with governments	Include strong limits and social justice in policies; reform important social institutions; change lifestyles and cultures through grassroots action	Adapt policies to include increases in efficiencies
Achievements/examples /implementations	Individual downshifting, transition Initiatives, eco-villages, policy reforms e.g. the 2019 Wellbeing Budget in New Zealand as a very first step	Individual downshifting, transition initiatives, eco-villages, Catalan Integral Cooperative, Rojava, Zapatistas	Individual downshifting, transition initiatives, eco-villages, policy reforms, e.g. the 2019 Wellbeing Budget in New Zealand as a first step	OECD and EU policies
Barriers	Lack of awareness among the public of limits to growth and alternatives; lack of research on these alternatives; changes could be too radical to be implemented; growth imperatives of states could be too much a barrier	Lack of awareness among the public of limits to growth and alternatives; lack of research on these alternatives; changes could be too radical to be implemented; barriers to grassroots action could be too high	Lack of awareness among the public of limits to growth and alternatives; lack of research on these alternatives; potential that problems cannot be solved within capitalism and centralised states	Priority still on economic growth
Alignment with dominant interests, systems and cultures	Low	Low	Low (Group 1) to medium (Group 2)	High

This table only provides a rough overview, focusing on the most obvious differences of the respective approaches. There are overlaps between them and considerable heterogeneity within each approach, e.g. eco-feminism and post-development overlap with eco-socialism and eco-anarchism.

The reformist group consists of heterogeneous approaches such as a-growth^80^, precautionary/pragmatic post-growth^52^, prosperity^42^ and managing^85^ without growth as well as steady-state economics^86^. These approaches have in common that they aim to achieve the required socio-ecological transformation through and within today’s dominant institutions, such as centralised democratic states and market economies^52,77^. From this position it often follows that current, socially vital institutions, such as the welfare state, labour markets, healthcare, pensions and others, need to be reformed to become independent from GDP growth^52^. Generally, bottom-up movements are seen as crucial, leading to value and cultural changes towards sufficiency^42,47^. Eventually, however, significant policy changes are proposed to achieve the necessary downshifting of consumption and production^42,77,86^ and/or the reduction of environmental impacts through decoupling^52,80^. These include, among others, stringent eco-taxes or cap-and trade systems, directed investments in green industries and public institutions, wealth redistribution through taxation and a maximum income, a guaranteed basic income and/or reduced working hours^42,77^. Although these policies already seem radical when compared to today’s policies, the proponents of reformist approaches are convinced that the transformation can be achieved in current capitalist economies and democratic states^42,77,86^.

The second, more radical, group disagrees and argues that the needed socio-ecological transformation will necessarily entail a shift beyond capitalism and/or current centralised states. Although comprising considerable heterogeneity^77^, it can be divided into eco-socialist approaches, viewing the democratic state as an important means to achieve the socio-ecological transformation^51,65^ and eco-anarchist approaches, aiming instead at participatory democracy without a state, thus minimising hierarchies^54,87^. Many degrowth approaches combine elements of the two, but often see a stronger role for state action than eco-anarchists^50,51,88^. Degrowth is defined here as “an equitable downscaling of throughput [that is the energy and resource flows through an economy, strongly coupled to GDP], with a concomitant securing of wellbeing“^59^^,p7^, aimed at a subsequent downscaled steady-state economic system that is socially just and in balance with ecological limits. Importantly, degrowth does not aim for a reduction of GDP per se, but rather accepts it as a likely outcome of the necessary changes^78^. Moreover, eco-feminist approaches highlight the role of patriarchal social relations and the parallels between the oppression of women and exploitation of nature^89^, while post-development approaches stress the manifold and heterogeneous visions of achieving such a socio-ecological transformation globally, especially in the global South^90^.

Degrowth advocates propose similar policy changes as the reformist group^50,80^. However, it is stressed that implementing these changes would most likely imply a shift beyond capitalism, e.g. preventing capital accumulation through dis-economies of scale and collective firm ownership, and thus require radical social change^59,62,91^. Eco-socialists usually focus more on rationing, planning of investments and employment, price controls and public ownership of at least the most central means of production to plan their downscaling in a socially sustainable way^65,77^.

Both groups agree on the crucial role of bottom-up movements to change culture and values, push for the implementation of these top-down changes and establish parts of the new economy within the old^47,50^. Finally, eco-anarchists do not view the state as a central means to achieve the socio-ecological transformation. Instead, they stress the role of bottom-up grassroots initiatives, such as transition initiatives and eco-villages, in prefiguring the transformation as well as cultural and value changes as a necessary precondition for wider radical change. With these initiatives scaling up, the state might get used to remove barriers and to support establishing a participatory-democratic and localised post-capitalist economy^54,77^.

In summary, there seems to be some strategic overlap between reformist and the more radical eco-anarchist and eco-socialist approaches, at least in the short term^77^. The question remains how these solution approaches help in overcoming the capitalist dynamics previously outlined, since here bottom-up and governmental action seem to be limited. It is important to recognise the pivotal role of social movements in this process, which can bring forward social tipping points through complex, unpredictable and reinforcing feedbacks^92,93^ and create windows of opportunity from crises^77,94^.

## New research directions

The evidence is clear. Long-term and concurrent human and planetary wellbeing will not be achieved in the Anthropocene if affluent overconsumption continues, spurred by economic systems that exploit nature and humans. We find that, to a large extent, the affluent lifestyles of the world’s rich determine and drive global environmental and social impact. Moreover, international trade mechanisms allow the rich world to displace its impact to the global poor. Not only can a sufficient decoupling of environmental and detrimental social impacts from economic growth not be achieved by technological innovation alone, but also the profit-driven mechanism of prevailing economic systems prevents the necessary reduction of impacts and resource utilisation per se.

In this context, the digital revolution—and more broadly the Fourth Industrial Revolution (FIR) with converging, step-change innovations in digital technology, artificial intelligence, Internet of Things, 3D-printing, biotechnology and nanotechnology—has been touted as an enabler of absolute decoupling through sheer exponential efficiency gains^95^. While digitalisation is already a key driving force in societal transformation, it has so far led to more consumption and inequality and remained coupled with the indirect use of energy and materials, therefore sustaining resource-intensive and greenhouse-gas growth patterns at the macro-economic level^17,96^. While the digital revolution undoubtedly increases labour productivity—demonstrated by individual leading businesses showing a strong productivity paradox—it remains to be seen whether the same is true for resource productivity, and this will depend on governance and regulation. Even if the FIR were to achieve absolute decoupling, this would come at a potentially high risk for privacy, liberty, data sovereignty, civic rights, security, equality and democracy^96,97^.

What is needed are convincing and viable solutions at the systems level that can be followed. We call for the scientific community across all disciplines to identify and support solutions with multidisciplinary research, for the public to engage in broad discussions about solutions and for policy makers to implement and enable solutions in policy processes. Based on the literature reviewed above we identify the following areas in need of further research. This list is not exhaustive or even fully conclusive, but rather meant to be the start of a continuous debate to frame future agendas of research and actions that need to be discussed and criticised.

### Research to advance basic academic understanding

Can inspiring visions for a sustainable life in prosperity, but within planetary limits and with less material affluence be formulated and demonstrated? How can fundamental changes in lifestyles of the affluent part of the human population be motivated and sustained?

The interface between materially downshifted lifestyles and the social environment (institutions, values, norms and governance) needs special attention. Which circumstances will allow for and support widespread shifts in lifestyles? What are the institutional, cultural and individual barriers to adopting lifestyle changes and how can they be overcome? What is the role of social groups, organisations and bottom-up movements? Can we learn from societies, e.g. indigenous and pre-industrial societies, which managed to live without economic growth?

So far, steady-state, degrowth or a-growth concepts have not practically been implemented on larger scales. Research on the environmental and social sustainability of these propositions is necessary (see e.g. ref. ^78^). Can a transition to reduced and changed consumption be achieved while at the same time keeping economic and social stability? What are the implications on work, employment and population growth? How can social security be maintained and equality be increased? What are the consequences for trade and for the global South in particular?

The scientific community should develop scenarios and possible pathways of strong sustainable consumption and production with upper and lower limits as suggested by the floor-and-ceiling framework, or sustainable consumption corridors^48,49,91,98^. These need to feature reduced physical throughput (possibly resulting in reduced GDP) and recomposing consumption^99^ with a simultaneous social reorientation of people, institutions and governments. Suitable indicators and scenarios based on interdisciplinary research need to be implemented to monitor progress^100^.

### Research on societal changes for citizens and communities

One first and immediate action anyone can take is to talk about overconsumption, i.e. current levels of consumption by most people in the global North, and how it is unsustainable and unethical or unjust. A wide debate in society, research and policy is necessary. Many people do not see themselves being part of either the problem or the solution, but look for governments, technology and/or businesses to solve the problem. The necessary alternative futures need to be discussed, envisioned and shared. It is important to create a sense of collective responsibility and action. Social sciences research and approaches can help by creating, providing and sharing concepts, experiences and platforms where public debates and dialogues take place. People who have already downshifted should be enabled to share their motivations and experiences to break through stigma and isolation, as would activists building a larger popular movement on climate action.

Research can identify the main issues to focus on primarily (flying, meat and dairy products, car driving, household sufficiency, etc.) and how cultures of sufficiency, care, solidarity and simplicity can be created. Individuals can downshift together as households and communities. Research can help to re-envision and reorganise cities to allow for shorter distances, closer communities, higher self-sufficiency, increased local place identity and more decentralised production, including that of food. More importantly, citizens can learn to engage as social actors to bring forward social tipping points^92^. These social tipping points include, for instance, removing fossil-fuel subsidies and investments, building decentralised energy generation or low-carbon cities. Such macro-efforts are clearly more important than individual ones, could help to address possible sufficiency rebound effects^47^ and thus deserve increased research attention and guidance.

Adding to this, as Smith et al.^93^ point out in reaction to Otto et al.^92^, it is crucial to ask “Who initiates deliberate, radical change in the collective interest?” and to recognise the pivotal role of social movements in this process.

### Research on governance

A number of concrete policy proposals for governance can be extracted from the literature (see also Cosme et al.^76^). All of these will need further scrutiny and research on their feasibility and implementation:

First, replace GDP as a measure of prosperity with a multitude of alternative indicators and be agnostic to growth. Expect likely shrinking of GDP if sufficient environmental policies are enacted. Research needs to advise on how best to monitor and report progress towards human and planetary wellbeing.

Second, empower people and strengthen participation in democratic processes and enable stronger local self-governance. Design governance and institutions to allow for social experiments, engagement and innovation. This could be trialled and organised e.g. through citizen assemblies or juries, as is demanded by Extinction Rebellion and already practised e.g. by Transition Initiatives or the Catalan Integral Cooperative^92^.

Third, strengthen equality and redistribution through suitable taxation policies, basic income and job guarantees and by setting maximum income levels, expanding public services and rolling back neoliberal reforms (e.g. as part of a Green New Deal^79^). Stronger regulation might be needed to ban certain products or ecologically destructive industries that have thrived on a legacy of vested interests, lobbying and state-supported subsidies.

Fourth, the transformation of economic systems can be supported with innovative business models that encourage sharing and giving economies, based on cooperation, communities and localised economies instead of competition. Research is needed to create, assess and revise suitable policy instruments.

And finally, capacity building, knowledge transfer and education—including media and advertising—need to be adapted to support local sufficiency projects and citizen initiatives.

## Data Availability

The authors declare that all data supporting this study are available within the paper (data sources for Fig. 1 have been provided in the figure legend).
